# Supplementary material for: Trauma Informed Child Welfare Systems—A Rapid Evidence Review
Source: Int J Environ Res Public Health. 2019 Jul 3;16(13):2365. doi: 10.3390/ijerph16132365 (PMC6651663; doi:10.3390/ijerph16132365)
Supplement: Supplementary file 1 [file ijerph-16-02365-s001.pdf]

Table S1. Search terms by database.

SCOPUS	TITLE-ABS-KEY (("trauma-inform*" OR "trauma inform*" OR "trauma-focus*" OR "trauma-base*" OR "trauma focus*" OR "trauma base*")) AND (LIMIT-TO (DOCTYPE , "ar") OR LIMIT-TO (DOCTYPE , " ip")) AND (LIMIT-TO (PUBYEAR , 2018) OR LIMIT-TO (PUBYEAR , 2017) OR LIMIT-TO (PUBYEAR , 2016) OR LIMIT-TO (PUBYEAR , 2015) OR LIMIT-TO (PUBYEAR , 2014) OR LIMIT-TO (PUBYEAR , 2013) OR LIMIT-TO (PUBYEAR , 2012) OR LIMIT-TO (PUBYEAR , 2011) OR LIMIT-TO (PUBYEAR , 2010) OR LIMIT-TO (PUBYEAR , 2009)) AND (LIMIT-TO (LANGUAGE , "English")
MEDLINE	("trauma-inform*" or "trauma inform*" or "trauma-focus*" or "trauma-base*" or "trauma focus*" or "trauma base*").ab. or ("trauma-inform*" or "trauma inform*" or "trauma-focus*" or "trauma-base*" or "trauma focus*" or "trauma base*").ti. or ("trauma-inform*" or "trauma inform*" or "trauma-focus*" or "trauma-base*" or "trauma focus*" or "trauma base*").kw. or ("trauma-inform*" or "trauma inform*" or "trauma-focus*" or "trauma-base*" or "trauma focus*" or "trauma base*").sh.<br>limit to (English language and yr="2009–Current" and journal article)
IBSS	(("trauma-inform*" OR "trauma inform*" OR "trauma-focus*" OR "trauma-base*" OR "trauma focus*" OR "trauma base*") OR ("trauma-inform* practice" OR "trauma inform* practice"))<br>Additional limits—Date: After 31 December 2008; Source type: Scholarly Journals
PSYCHINFO	("trauma-inform*" or "trauma inform*" or "trauma-focus*" or "trauma-base*" or "trauma focus*" or "trauma base*").ab. or ("trauma-inform*" or "trauma inform*" or "trauma-focus*" or "trauma-base*" or "trauma focus*" or "trauma base*").ti. or ("trauma-inform*" or "trauma inform*" or "trauma-focus*" or "trauma-base*" or "trauma focus*" or "trauma base*").mh.<br><br>limit 1 to (English language and yr="2009–Current")<br>limit 2 to journal article
ERIC	TI ((("trauma-inform*" OR "trauma inform*" OR "trauma-focus*" OR "trauma-base*" OR "trauma focus*" OR "trauma base*"))) OR AB ((("trauma-inform*" OR "trauma inform*" OR "trauma-focus*" OR "trauma-base*" OR "trauma focus*" OR "trauma base*"))) OR KW ((("trauma-inform*" OR "trauma inform*" OR "trauma-focus*" OR "trauma-base*" OR "trauma focus*" OR "trauma base*")))<br>Limiters—Date Published: 20090101–20181231; Journal or Document: Journal Article (EJ); Language: English
WOS	(TS=("trauma-inform*" OR "trauma inform*" OR "trauma-focus*" OR "trauma-base*" OR "trauma focus*" OR "trauma base*")) AND LANGUAGE: (English) AND DOCUMENT TYPES: (Article)<br>Indexes=SCI-EXPANDED, SSCI Timespan=2009–2018

Table S2: State-wide implementation initiatives.

Country and Setting	TIC Approach and Components
Country—USA<br><br>Setting—TIC implementation in child welfare system in five states	**Five state-wide projects implementing trauma screening for children in the child welfare system (CWS)**<br><br>Colorado<br>• Goal was to provide universal screening for all children aged birth to 18 involved in the CWS who had an open case for ongoing services, including voluntary and court-ordered child protective services (CPS) involvement (excluding children seen only in intake/investigations). Individual consultation was provided.<br><br>Connecticut

	• Aimed to screen all children aged 6 to 17 who were entering the care of the CWS following removal from the family of origin.<br>Massachusetts<br>• Plan to screen all children aged birth to 18 following a CPS report that has been flagged for further assessment.<br>Montana<br>• The implementation plan was to screen all children who were in contact with the Bureau of Indian Affairs CWS.<br>North Carolina<br>• Aimed to screen children from birth to age 18 entering foster care. Screening children in other units (e.g., intake/investigations) was optional.

Author/Reference	Evaluation Design	Results and Limitations
Lang, Ake, Barto, Caringi, Little, Baldwin, Sullivan, Tunno, Bodian, Joy Stewart, Stevens and Connell [37]	**Design:** Description of five state-wide initiatives to implement trauma screening for children in the child welfare system. Relies primarily on administrative data, as well as a staff surveys/interviews in some sites.<br><br>**Measures:** Colorado, the Child Trauma Assessment Centre (CTAC) screen (Henry et al; Connecticut, the Child Trauma Screen (CTS): Massachusetts, the NCTSN-adapted Child Welfare Referral Tool, later incorporated into the Family Assessment and Action Plan; Montana, the CTAC screen: North Carolina, the 6- and 11-question versions of the Project Broadcast Screening Tool (Sullivan et al.).	**Findings:**<br>Colorado: In the initial 16 months of implementation, 1315 children/youth were part of an open CPS case. Of these, 697 (53%) were screened by 180 caseworkers and 39 supervisors.<br>- Connecticut: A total of 601 children were screened with the CTS between November 2014 and June 2016 by 11 clinicians.<br>- Massachusetts: Of 42 agencies, the average rate of screening increased from baseline (40.3%) to follow-up (75.0%).<br>- Montana: The initial numbers of children screened has been low, with only ten or fewer children screened at each site.<br>- North Carolina: Over the course of 36 months (January 2013 to December 2015), child welfare workers in the twelve Project Broadcast counties completed a total of 9714 trauma screens (66% Assessment/Investigation, 26% Foster Care/Out-of-Home Placement, 6% In-Home, 1% Other) representing 6651 unique children (some children were re-screened).<br>Overall, findings indicate that implementation strategies varied considerably but that screening generally resulted in identification of high rates of trauma exposure, trauma symptoms and service referrals.<br>- Noted that implementation of trauma screening in each of the five CWSs has been a somewhat lengthy and challenging process in comparison with other activities such as EBT dissemination, training staff in childhood trauma.<br>Screening was generally perceived favourably by child welfare workers and mental health professionals. However, wide variations were observed in the number of children screened, suggesting that more research is needed to identify optimal strategies.<br><br>**Limitations:** primarily descriptive and process orientated, evidence about the effects of screening on service referrals, access to treatment or child welfare outcome is still needed.

Country and Setting	TIC Approach and **Components**
Country—USA<br>Setting—State-wide initiative in Massachusetts	**Massachusetts Child Trauma Project**<br>• Basic and advanced child trauma training with Child Welfare (CW) staff using the National Child Traumatic Stress Network (NCTSN) Child Welfare Training Toolkit<br>• Workshops for foster parents (Caring for Children Who Have Experienced Trauma: A Workshop to Resource Parents)<br>• State-wide dissemination of three trauma treatments with empirical support via community based mental health organizations: ARC, child-parent psychotherapy and trauma-focused cognitive–behavioural therapy.<br>• Dissemination involved comprehensive training and consultation in the form of a learning collaborative (LC) model which brings together senior managers, clinical supervisors, clinicians and data managers who commit to a one-year learning period and involved face-to-face learning sessions and intensive EBT consultation.<br>• Creation of Trauma-Informed Leadership Teams (TILTs), focused on installing and supporting a structure for trauma informed care systems integration at the community level. They rely on leadership by CW management and participation by social workers, service users, mental health providers and other community service providers and stakeholders.

Author/Year	Evaluation Design	Results and Limitations
Fraser, Griffin, Barto, Lo, Wenz-Gross, Spinazzola, Bodian, Nisenbaum and Bartlett [25]	**Design:** Preliminary implementation evaluation—used a multi-source, multi-method approach including surveys, records reviews and individual child assessments to measure both process and outcome. Participants included 24 Area Office Directors, 153 clinicians, 645 child welfare and mental health workers involved in training and 17 TILTS.<br>**Measures:** The Trauma System Readiness Tool was administered to Area Office Directors (completion rate = 83%). The Evidence-Based Practice Attitudes Scale (EBPAS) and the Trauma-Informed System Change Instrument (TISCI) measures were administered to the clinicians (response rate = 79.7%). A training evaluation was completed by 645 child welfare and mental health workers (response rate = 59%) and all 17 Area Office TILTs completed a self-assessment at the end of year one.	**Findings:**<br>- Responses to the EBPAS were overall positive and suggested that clinicians did not have a negative attitude towards evidence-based practice.<br>- Scores on the TISCI were similarly positive, indicating that at the individual level, clinicians had a strong intention to consistently engage in trauma-informed practice.<br>- 80% of workers who completed the training evaluation reported being satisfied or very satisfied with the training.<br>- Feedback from the TILT teams pointed to the championing role of Area Office managers as the critical factor to successful teams; another key factor was the comfort level in engaging members of the community on their team.<br>- EBT implementation progress: at the end of the first year of implementation, 298 children were enrolled in an EBT.<br>**Limitations:** lack of child outcomes and baseline measures.
Bartlett, Barto, Grifflin, Goldman Fraser, Hodgdon and Bodian [26]	**Design:** interviews with 32 TILT leaders and 25 senior leaders in learning collaboratives (LCs); single group pre-test/post-test training evaluation provided to 190 community mental health practitioners with one year	**Results:**<br>- Interviews pointed to TILTS as key structures for TIC systems integration participation in evidence-based treatment (EBT). Learning collaboratives were linked to improvements in trauma informed individual and agency practices. Scarce resources

	follow up and 81% retention; baseline assessment and six-month follow up of 326 children referred to treatments.<br><br>**Measures:** training measured via Trauma-Informed System Change Instrument, clinical assessment measured PTSD symptoms of children aged 7–18 using the Posttraumatic Stress Disorder Reaction Index (UCLA PTSD-RI), and for behaviour of children aged 6–18 we used the Child Behaviour Checklist (CBCL).	for trauma-related work in the CW agency and few services providing EBTs to young children emerged as barriers to TIC.<br>- Training evaluation found significant changes in perceptions of trauma-informed agency policy as well as perceptions of individual practices.<br>- After approximately six months of EBT treatment, children had fewer post traumatic symptoms and behaviour problems<br><br>**Limitations:** hard to attribute specific factors to outcomes, training outcomes largely self-reported.
Barto, Bartlett, Von Ende, Bodian, Noroña, Griffin, Goldman Fraser, Kinniburgh, Spinazzolo, Montagna and Todd [17]	**Design:** comparison of outcome for 55,145 children who received the MCTP intervention (Cohort I; northern and western areas of the state) and 36,108 who did not (Cohort II; Boston and southern areas of the state) during the first year of implementation.<br><br>**Measures:** child welfare administrative data on child maltreatment, out-of-home placements, and adoption: the total number of maltreatment reports; the total number of substantiated maltreatment reports; the total number out-of-home placements.	**Findings:**<br>- Children in the intervention group had fewer total substantiated reports of maltreatment, including less physical abuse and neglect than the comparison group by the end of the intervention year.<br>- However, children in the intervention group had more maltreatment reports (substantiated or not) and total out-of-home placements than did their counterparts in the comparison group—it is possible that this was related to increased surveillance and reporting of maltreatment and placement issues by MCTP's trained child welfare caseworkers and EBT providers.<br>- Assignment to MCTP, however, was not associated with an increase in kinship care or adoption.<br>- Overall, the results are promising in reinforcing the importance of mobilizing communities toward improvements in child-welfare service delivery.<br><br>**Limitations:** Study children were not randomly assigned to intervention; intervention and control children differ in their background characteristics. However, this was accounted for by conducting an inverse probability of treatment weighted analysis.

Country and Setting	TIC Approach and Components
Country—USA<br><br>Setting—State-wide family service, case workers/child protection social workers and other staff in Arkansas	**Arkansas Initiative**<br>• Phase One—targeted all area directors and regional and local supervisors in the state's child welfare system, who attended one of ten, two-day, regional trainings using National Child Traumatic Stress Network (NCTSN) content. This also involved a train-the-trainer component (see Kramer et al.)<br>• Phase Two—targeted all front-line child welfare workers over the course of a year and involved a one-day workshop led by social workers. It was designed to increase awareness of the effects of trauma on children; promote evidence-based screening, assessment and treatment and coordinate care with other service agencies. The training was focused on nine essential elements:<br>- maximize the child's sense of safety;

	- assist children in reducing overwhelming emotions;<br>- help children make new meaning of their trauma history;<br>- address the impact of trauma and subsequent changes in the child's behaviours, development and relationships;<br>- coordinate services with other agencies;<br>- utilize comprehensive assessment of a child's trauma experience and the impact on the child's development and behaviour to guide services;<br>- support and promote positive and stable relationships in the life of the child;<br>- provide support and guidance to the child's family and caregivers;<br>- manage professional and personal stress.<br>• As part of the training child welfare work, workers were also asked to create an action plan for using trauma-informed child welfare practices based on the "Bringing It Back to Work" tool available in The Child Welfare Trauma Training Toolkit<br>• Trauma-informed training for the child welfare system was conducted following dissemination of trauma-focused cognitive behavioural therapy (TF-CBT) to more than 150 mental health professionals across the state to maximize capacity for assessment and treatment referrals once child welfare workers were better informed about the effects of trauma on children.

Author/Year	Evaluation Design	Results and Limitations
Kramer, Sigel, Conners Burrow, Savary, and Tempel [29]	**Design:** pre-test/multiple post-tests evaluation of training with 102 DCFS leaders (Follow-up:immediately after training then at three months with 78% retention).<br><br>**Measures:** Knowledge of trauma-informed practice and self-reported use of trauma-informed practices, measured via questionnaire developed by intervention developers.	**Findings:**<br>- Significant increases in knowledge about trauma-informed practice between pre-test and immediately post-test.<br>- Significant increases in self-reported use of trauma-informed practices between pre-test and three months follow-up.<br><br>**Limitations:** no control group, short follow up, based on self-reporting.
Conners-Burrow, Kramer, Sigel, Helpenstill, Sievers & McKelvey [30]	**Design:** Pre-test/multiple post-tests evaluation of training with child welfare staff (n = 438, follow up immediately after training, retention 93%) and a random sample of child welfare staff (n = 161, three-month follow up, retention 88%). Additionally, half of the child welfare staff who were followed at three months were asked to complete a longer interview that asked about their success in implementing the action steps listed on their individualized plan developed at the end of training (n = 68).<br><br>**Measures:** knowledge of trauma-informed practice and self-reported use of trauma-informed practices, measured via questionnaire developed by intervention developer.	**Findings:**<br>- Knowledge of trauma-informed practice increased significantly between pre-test and post-test, as did self-reported changes in practice, although effect sizes were small when it came to direct support services for children and moderate for indirect support services.<br>- 43.3% reported that they were able to fully implement the strategy identified at training, while another 43.3% were partially implemented and 13.4% were unable to implement the strategy.<br><br>**Limitations:** no control group, short follow up, based on self-reporting.

Country and Setting	TIC Approach and Components

Table S3: Organisational/agency implementation initiatives.

Country—USA<br><br>Setting—Staff in child welfare agencies in Connecticut	The Connecticut Collaborative on Effective Practices for Trauma (CONCEPT)<br><br>• Creation of a core team and subcommittee to guide trauma informed systems change.<br>• Development of a cohort of 40 "trauma champions" who organized one in-service training about trauma every month.<br>• State-wide mandatory preservice and in-service trauma training for child welfare staff, involved implementation of the NCTSN Child Welfare Trauma Training Toolkit—Training was provided to 487 managers and supervisors in the spring of 2013 and to 1164 caseworkers and clinical staff in the fall of 2014.<br>• "Worker wellness" (i.e., staff support) teams created and quarterly trainings in self-care provided.<br>• Revision of agency policies for alignment with trauma-informed practice.<br>• Training in trauma-focused cognitive behavioural therapy for community-based service providers.	
**Author/Year**	**Evaluation Design**	**Results and Limitations**
Lang, Campbell, Shanley, Crusto, and Connell [31]	**Design:** Pre-test/post-test with a stratified random sample of 230 staff at a child welfare agency. Follow-up: two years.<br><br>**Measures:** Perceptions of individual and agency capacity to provide trauma-informed care, measured via Trauma System Readiness Tool. Perceptions of individual and agency capacity to provide trauma-informed care	**Findings:**<br>- perceptions of individual and agency capacity to provide trauma-informed care increased significantly for 11 of the 12 domains.<br><br>**Limitations:** response rate less than 45% for pre-test and post-test.
**Country and setting**	**TIC Approach and Components**	
Country—USA<br><br>Setting—Child welfare agencies in Washington State	Creating Connections—a five-year project in Washington State.<br><br>Training as part of a large-scale initiative to integrate trauma-informed and trauma-focused practice in Child Protective Service delivery.<br><br>Washington State Department of Social and Health Services—Children's Administration (DSHS-CA) already employed a robust screening strategy called the Child Health and Education Tracking (CHET) prior to implementation. The state-wide implementation of the Screen for Child Related Anxiety Emotional Disorders within CHET was initiated in July 2014. In addition, a new program was implemented to provide ongoing screening (called Ongoing Mental Health (OMH) screening program).<br><br>Training<br>- An initial training was conducted with Child Health and Education Tracking supervisors, followed by a two-hour training to all state-wide CHET/OMH staff. Training included how to gather, interpret and share screening data with the child welfare professional.<br>- Three-hour training required for newly hired child welfare professionals (regional core training (RCT)) and a six-hour, in-depth, focused training for any child welfare professional (In-Service Training (IST)).	
**Author/Year**	**Evaluation Design**	**Results and Limitations**

Kerns, Pullmann, Negrete, Uomoto, Berliner, Shogren, Silverman & Putnam [28]	**Design:** Pre-test/multiple post-tests evaluation of training with 44 CHET/OMH staff and 71 child welfare staff at a child welfare agency. Follow-up: Immediately after training and at six months with 70.5% retention with CHET/OMH staff and immediately after training with child welfare professionals .<br><br>**Measures:** self-reported knowledge and skills gained via questionnaire developed by intervention developers.	**Results**<br>- CHET/OMH staff knowledge and skills for administering the PSC-17 increased significantly and was retained at six-months follow-up.<br>- CHET/OMH staff knowledge and skills for administering the SCARED increased significantly and retained at six months follow-up.<br>- For the RCT, self-reported competency scores on nearly all items, including isotem score, significantly improved from pre- to post-training.<br>Self-reported competency scores on all items on the IST significantly improved from pre- to post-training, including total item means.<br>**Limitations:** no longer-term follow up for child welfare staff training, all findings based on self-reporting.

Country and Setting	TIC Approach and Components
Country—USA<br><br>Setting—Child welfare agencies from nine Michigan counties	Michigan Children's Trauma Assessment Center (CTAC)<br><br>The primary purpose was to develop a framework and protocol for implementing Trauma Informed Child Welfare Systems (TICWSs) at the local community level in a "bottom-up" or grassroots approach<br><br>TICWSs Partnership Model:<br>• CTAC provided training to community members—members become interested in developing in their community and become champions.<br>• CTAC engage in community discussions and potentially invest—community members engage local leaders to discuss possibilities with CTAC.<br>• CTAC meet with local leaders to assess capacity to take on initiative —leadership discusses commitment to project.<br>• CTAC make a commitment to the community based on engagement and readiness – leaders plan community initiative.<br>• CTAC and leadership identify areas to target using training, consultation, assessment capacity and treatment capacity.

Author/Year	Evaluation Design	Results and Limitations
Henry, Richardson, Black-Pond, Atchinson [32]	**Design:** Qualitative and quantitative evaluation of initiative involving professionals and resource parents from welfare agencies in nine counties. This include baseline evaluation of the current state of trauma-informed practices and readiness to change, and one year follow up (n = 631); as well as eight interviews of key personnel and secondary data (court neglect/abuse file) reviews (53 files representing 112 children).	**Findings:**<br>- Post-test results after one year revealed a statistically significant increase in the extent that policy had become more trauma-informed.<br>- Agency practice likewise showed statistically significant improvement.<br>- Six key areas for implementation emerged as critical in the development of a TICWS;<br>1) Development of champions: Champions were identified through their own interest and initiative to mobilize resources and bring together community

	**Measures:** Trauma-Informed System Change Instrument administered to professionals and parents to measure change.	members central to system change. Their efforts were simultaneously supported by CTAC participation in leadership meetings, reinforcing interest and momentum for change.<br>2) Screening and identification of trauma in children: 964 screens were anonymously collected for children ages 0 to 17, primarily through DHS workers, resource parents and school personnel. Results were used to raise awareness of the prevalence of children who have been traumatized and its impact in their community—Two project communities have used results to secure funding from local foundations for the development of trauma assessment centres. Approximately a third of Michigan's local Community Mental Health agencies adopted the TSC for screening children at intake, with one agency completing 4500 screens.<br>3) Comprehensive assessment of the impact of trauma: To date, CTAC has trained three teams of professionals (out of the nine communities) in a transdisciplinary neurodevelopmental assessment protocol. The teams are comprised of professionals from mental health, occupational speech/language, and the medical field.<br>4) Development of a cadre of community therapists (public and private): In Year 1 of the grant, 29 clinicians from three county systems were trained in Trauma-Focused Cognitive Behavioural Therapy (TF-CBT) and/or Real Life Heroes using a learning collaborative methodology. This involved two-day training with a year-long consultation protocol including monthly phone consultation and quarterly in-person consultations. During Year 3 of the grant, a second TF-CBT training was provided for 22 therapists with 19 continuing with follow-up consultation. In each community, additional training for clinicians and school social workers was provided to introduce phase-based and trauma-informed interventions appropriate in their setting—this included psychoeducation, self-regulation skill building, trauma processing and safety planning (trigger management). During 2010 over 230 children received TF-CBT from project trained therapists.<br>5) Establishment of Common Language Using Trauma-Informed Instruments: Specific trauma trainings provided to courts, schools, DHS, CMH, medical personnel and caregivers to infuse trauma into agency and interagency discussion of children. To further infuse trauma language CTAC developed a trauma-informed Court Report Checklist. Two years following implementation of the CRC in the first pilot community, 100% of the cases had a CRC submitted by the DHS worker to the judge prior to court hearings.<br>6) Use of Trauma Knowledge in Decision-Making for Children: For legal decision-making process, a document highlighting the Essential Elements for attorneys was developed. Trauma-informed child welfare decision-making was identified as one of the greatest needs and most significant challenges

		within pilot communities and developed training to address issues of secondary traumatic stress and decision-making regarding removal of children from biological parents and placement changes.<br><br>**Limitations:** no measurement of child outcomes, change based on self-reporting.
**Country and Setting**	**TIC Approach and Components**	
Country—USA<br><br>Setting—Foster care and adoption	New Hampshire Adoption Preparation and Preservation/Partners for Change Project<br><br>Part of a 5-year, federally funded project to install evidence-based, trauma-informed practices into the child welfare and mental health systems in one US state. Aimed to develop, and rigorously evaluate, new practices targeted specifically for a pre and post adopt population. These practices included: 1) universal, flexible, and ongoing child screening and assessment specific to the pre and post adoption population of children; 2) case planning strategies specific to the target population; 3) service array reconfiguration to upscale evidence-based treatments specific to the needs of pre and post adoptive families; 4) ongoing family assessments for all resource families and a corresponding child matching process to ensure fit when placing children in pre adoptive homes; and 5) training for resource parents and DCYF staff working with pre and post adoptive families related to trauma and behavior management.	
**Author/Year**	**Evaluation Design**	**Results and Limitations**
Barnett, Cleary, Butcher and Janowski [18]	**Design:** Online and postal survey of licensed foster families, formerly licensed families and adoptive families from the past 10 years of records in one US state (not specified). Aimed at examining whether foster and adoptive parent perceptions of the quality of trauma-informed child welfare and mental health services moderate the relationship between children's behavioural health needs and parent satisfaction and commitment. Family units totalling 1206 were identified and 512 responded (42%: fostering only (n = 168), adoptive only (n = 215), fostering and having adopted (n = 66).<br><br>**Measure:** survey instrument designed by researchers.	**Findings:**<br>- Trauma-informed mental health services (but not child welfare services) moderated the relationship between child behavioural health needs and foster parent (but not adoptive parent) satisfaction and commitment.<br>- There was a significant interaction between child behavioural health needs and parent satisfaction and commitment (at low levels) of trauma-informed mental health services suggesting that these can buffer them against low satisfaction and commitment, and thereby, potentially improve placement stability.<br><br>**Limitations:** No standardised or validated measures. Based on adoptive parent and foster carer subjective perception of child behaviour problems and the quality of trauma informed mental-health and child welfare services. Low response rate.

Country and Setting	TIC Approach and **Components**
Country—USA<br><br>Setting—Training of mental health professional with child welfare affiliated professionals working in the area of adoption across 16 states	Training for Adoption Competency (TAC)<br><br>Developed by the Centre for Adoption Support and Education (CASE) in Petersburg, Virginia in response to an identified unmet need for quality, adoption-competent mental health services for adoptive families<br><br>The TAC is a fully manualised programme which has been replicated with 59 cohorts of more than 900 professionals in 16 states. It involves:<br>• A 12-module curriculum culminating in a final project requiring integration of learning and application to practice,<br>• Six-monthly clinical case consultation sessions, facilitated by expert clinicians and designed to reinforce the transfer of learning into clinical practice,<br>• a robust trainer credentialing and support process featuring selection in accordance with prescribed qualifications, a week-long in-person orientation, debriefing calls after modules that are informed by participant feedback and fidelity observations, and ongoing supportive technical assistance and<br>• an ongoing multicomponent evaluation examining training delivery, effectiveness and outcomes.

Author/Year	Evaluation Design	Results and Limitations
Atkinson and Riley [19]	**Design:** Evaluation of training fidelity using observation and feedback and pre/post-test evaluation of training outcome which involving 855 participants including mental health professionals, public and private mental health agencies, adoption-specialty organizations, family service agencies, private practices, child welfare agencies residential treatment facilities and other settings. Training outcomes assessed mid training and end of training—timing not specified. Reference to control group but details not provided<br><br>**Measures:** Training outcomes measured/assessed via mid training and end of training surveys of participants and a self-assessment of adoption competency administered at the conclusion of the modules as a retrospective pre- and post-assessment. Training fidelity assessed using fidelity observations and feedback from participants and trainers to assess the quality and relevance of training and the fidelity of curriculum delivery.	**Findings:**<br><br>Training Delivery<br>- More than 300 fidelity observations of training delivery across 59 cohorts confirm full delivery, with fidelity, of nearly 100% of all content of all modules.<br><br>Training Effectiveness<br>- TAC participants experienced an average gain in pre- to post-scores of 46.08 points, while those in the control groups of comparably qualified professionals experienced a gain of only 1.58 points.<br>- There was not a statistically significant difference in test scores between participant and control groups at pre-test.<br>- There was a significant interaction between the training and time on test scores.<br><br>Changes in practice<br>- Based on 1148 responses containing 4928 separate narrative descriptions of the ways practices were influenced by the training, all TAC participants reported change in at least two of the six defined aspects of practice, 59.88% reported change in all five aspects at the individual clinician level and 51.75% reported that TAC influenced the procedures, programming and/or services in their organization.

		**Limitations:** Measures of training outcomes and changes in practice primarily self-report, details of controls not provided and sample size not always clear.

Country and Setting	TIC Approach and Components
Country—USA<br><br>Setting—Preschool and school-aged children in the child protective system treated through the Alaska Child Trauma Centre	**Attachment, Regulation, Competency (ARC) Model**<br><br>Focuses on:<br>- Caregiver management of affect<br>- Attunement<br>- Consistent response<br>- Routines and rituals<br><br>Paper focuses on specific case examples rather than agency wide approaches.

Author/Reference	Evaluation Design	Results and Limitations
Arvidson Kinniburgh, Howard, Spinazzola, Strothers, Evans & Blaustein [19]	**Research design:** naturalistic pre-test, post-test programme evaluation of treatment outcomes and placement stability in 93 children treated using ARC model (only 26 completed the intervention).<br><br>**Measures:** Agency data and clinical assessments using Trauma Symptom Checklist Alternate Version, the UCLA PTSD Index for DSM IV and the Child Behaviour Checklist (CBCL) used with all children. Administered at baseline, at three-month intervals and at discharge.	**Findings:**<br><br>- The average drop in CBCL scores for children completing treatment was 19 points.<br>- 90% children moved to permanent placements compared to usual 40%.<br><br>**Limitations:** no comparison group so not clear how it compares to treatment as usual; small numbers.

Country and setting	TIC Approach and Components
Country—USA<br><br>Setting—Staff at five Child Advocacy Centres in Florida	**Child Advocacy Centres Florida**<br><br>Half day based on National Child Traumatic Stress Network trauma-informed training. It included seven Essential Elements:<br><br>1. Maximize physical and psychological safety for the child.<br>2. Identify trauma-related needs of children.<br>3. Enhance child well-being and resilience.

	4. Enhance family well-being and resilience.<br>5. Enhance the well-being and resilience of those working in the system.<br>6. Partner with youth and families.<br>7. Partner with agencies and systems that interact with children and families.

Author/Year	Evaluation Design	Results and Limitations
Kenny, Vazquez, Long & Thompson [36]	**Design:** Pre-test/multiple post-test design to evaluate training with 203 staff who participated in training between August 2013 and October 2014. Follow-up: Immediately post training and one year.<br><br>**Measures:** knowledge about trauma-informed care via questionnaire developed by intervention developers.	**Findings:**<br><br>- Knowledge about trauma-informed care increased significantly between pre- and immediately post-training and was retained at after one year.<br><br>**Limitations:** 12% pre-test/one-year post-test follow-up retention.

Country and setting	TIC Approach and **Components**
Country—USA<br><br>Setting—Family Preservation Services in Denver	**Indian Child Welfare Family Preservation Services**<br><br>The model encompasses both systemic and direct practice efforts that assist families facing multiple challenges in creating a nurturing and more stable family life. The components of direct practice interventions include:<br>• strengths-based, culturally appropriate, and trauma-informed intake and family assessments and<br>• concentrated and family-focused case management services and referrals for material resources (e.g., housing, food, legal, transport, etc).<br><br>The components of systemic interventions include:<br>• establishment of protocols for early identification of American Indian families and children within the child welfare system and<br>• referral of families for culturally appropriate family preservation services.

Author/Year	Evaluation Design	Results and Limitations
Lucero and Bussey [20]	**Design:** Evaluation of three years of family preservation services which served 73 families and 179 children over three years. Involved two projects: the Rocky Mountain Quality Improvement Centre (RMQIC) program and a second project, funded by a grant from the Colorado Department of Human Services' Statewide Strategic Use Fund (the SSUF program).<br><br>**Measures:** Family functioning (North Carolina Family Assessment Scale (NCFAS), the Family Assessment Device, and the Parent Behaviour Inventory). Child safety was	**Findings:**<br><br>- A positive trend was seen in Family Safety for those families in the RMQIC program.<br><br>- Families in the SSUF program showed significant positive change in the area of Environment, and positive trends in the areas of Caregiver Capabilities, Family Safety and Child Well-Being.<br><br>- There were no re-reports during program services or within six months for any of the 49 families served by the RMQIC project, and one new report within six months after services

	measured directly by re-reports to CPS and indirectly through improvement on the Family Safety subscale of the NCFAS-AI (American Indian version of the NCFAS).	for the 24 families served by the SSUF project. This compares favourably with national re-report rates.<br><br>- In the RMQIC project, 81% of families had their children preserved in the home, returned (if out-of-home care was used), or placed with extended family members.<br><br>- In the SSUF project, 96% of families were preserved with children either at home with parents (the most common result) or with extended family members.<br><br>**Limitations:** no previous program baseline data presented and comparison only by national averages.

Country and Setting	TIC Approach and **Components**
Country—USA<br><br>Setting—Social services agencies (setting unspecified)	**Sanctuary Model**<br><br>Based on the concept of therapeutic communities, the Sanctuary Model is described as a theory-based, trauma-informed, value-driven, evidence-supported whole-culture approach that has a clear and structured methodology for creating or changing an organizational culture. It is designed to facilitate the development of structures, processes, and behaviours on the part of staff, clients and the community as a whole that can counteract the biological, affective, cognitive, social and experiential wounds suffered by the victims of traumatic experience and extended exposure to adversity.<br><br>The four core elements of the Sanctuary Model are (a) trauma theory; (b) the Seven Commitments—nonviolence, emotional intelligence, democracy, open communication, social responsibility, social learning and growth and change; (c) S.E.L.F.—an acronym for the organizing categories of safety, emotion management, loss and future, which is used to formulate plans for client services or treatment as well as for interpersonal and organizational problem solving; and (d) the Sanctuary Tool Kit, a set of 10 practical applications of trauma theory, the Seven Commitments and S.E.L.F., all of which are used by all members of the community at all levels of the hierarchy and reinforce the concepts of the model.<br><br>Implementation process consisted of:<br>• initial five days training for key leaders and<br>• leaders returning to the agency and forming Core Team of representatives across all levels and departments who are primary change agents.

Author/Year	Evaluation Design	Results and Limitations
Middleton, Harvey and Esaki [35]	**Design:** Qualitative design to evaluate implementation of model (n = 5, from two agencies).	**Findings:**<br><br>Leaders reported:<br>- Experiences that were compatible with four tenets of the "transformational leadership model".<br>- Modelling behaviours which promoted the trauma-informed model, of being a champion for TIC.

	**Measures:** single semi-structured telephone/skype interviews with 24 standardized questions.	- The need for a shared belief with staff in the mission and vision of the organization and the need to include staff from the very beginning.<br>- The importance of including staff in discussions showed respect for staff voices and participation.<br>- The importance of staff self-care, of being supportive of their needs and showing concern for their well-being, work–life balance and physical and emotional safety<br><br>**Limitations:** small sample, subjective, self-report, no outcome measures.
**Country and Setting**	**TIC Approach and Components**	
Country—USA<br><br>Setting—Training for family home visitors and parent educators in Kansas and Iowa	**Lemonade for Life**—pilot of training and routine use of ACE questionnaire.<br><br>A training initiative developed to help professionals who work directly with families understand how to use ACE research as a tool to build hope and resilience.<br><br>Training includes:<br>• A three-hour online ACEs module, a six-hour in-person training, and a ninety-minute coaching call approximately six weeks after the training.<br>• After the three-hour online training, participants are asked to complete a pre-test comprised of questions about the participant's understanding of their own ACE score and ACEs in general and items from the Hope Scale.<br>• Core elements of the six-hour training include: 1) education and reflection on ACEs, including the home visitor's own ACEs score; 2) intentional practice and action; and 3) hope theory and ways to foster hope and resilience.<br>• Participants receive materials that can be used with families during home visits, including The Amazing Brain handouts (Chamberlain); a Strengthening Resiliency Plan; and a Hope Map (Lopez).<br>• During the training, home visitors prepare their own script for introducing the ACEs questionnaire, as well as guidance on what to say and what not to say to families. Participants also receive a checklist to help them assess whether a family is ready and the timing is right to administer the ACEs questionnaire and have a conversation about the results.	
**Author/Year**	**Evaluation Design**	**Results and Limitations**
Counts, Gillam, Perico and Eggers [34]	**Design:** Pre/post-test evaluation of pilot training with 24 home visitors and parent educators in Kansas and Iowa. Seventeen completed all program phases and participants completed all phases and follow up was approximately six weeks after training completion. Focus groups with participants—number not stated.<br><br>**Measures:** Survey data, included items from the Hope Scale (Lopez) and Lemonade for Life-specific questions including: demographic information; participant experiences with ACEs personally and professionally; participant perceptions of using ACEs in work with families. Focus groups utilised a semi-structured guide asking	**Findings:**<br><br>- Mean scores increased from pre to post in several areas: understanding how early experiences influence life course; home visitors' knowledge of and self-reflection on their own ACEs score; and knowing where to refer someone who is struggling with ACEs. The mean score on both hope items "I have the power to make my future better" and "I make others feel excited about the future" decreased from pre to post.<br>- Qualitative data identified three major themes expressed by participants following the Lemonade for Life training and use of ACEs with families: 1) engagement increased between home visitors and families; 2) families gained an understanding of the connection between life choices and ACEs; and 3) the training and materials were easy

	questions about participant reactions to the training, use of materials with families, and additional questions or needs around the use of ACEs with families.	for the home visitors and parent educators to understand and provided tangible tools for use in work with families.<br><br>**Limitations:** small sample, largely self-reported, only portions of the Hope Scale were used, no family outcomes.

Country and Setting	TIC Approach and **Components**
Country—USA<br><br>Setting—KVC Kansas—an organisation that provides out-of-home care to children served by the Kansas Department for Children and Families in the Kansas City Metropolitan and East Kansas regions.	**KVC Kansas**—Trauma Systems Therapy (TST) Model<br><br>System wide implementation of TST in an organization providing out-of-home care to children:<br>1. Staff training: formal TST trainings for all staff and supervisors were conducted in early 2012. Training included an in-person training session (with overflow staff receiving training via video conference) and a follow-up training on the use of assessments—the UCLA-Post Traumatic Stress Disorder (PTSD)-Reaction index in particular. Attendees were required to complete assigned readings of the book written by TST developers (Saxe et al.) and participate in "book reviews" (conducted via WebEx) prior to the in-person training.<br>2. Coaching, mentoring and continuous quality improvement: All staff were afforded coaching and mentoring in TST through supervision. During weekly case consultations, supervisors mentored staff and provided instruction on TST. KVC, in conjunction with the TST developers, constructed fidelity measures that were administered quarterly by supervisors. Other efforts to ensure staff received support included the development of a "sustainability" team consisting of 12 staff members who provided oversight to the TST implementation effort. Additionally, staff in the residential and hospital program developed a number of different training "boosters".<br>3. Foster parent training—In addition to providing opportunities for foster families to attend formal training sessions, KVC developed an online training component, aligned with a specialized workbook, which families could use in their own homes.<br>4. Case consultation calls—Regularly scheduled conference calls involving all members of the child's team (case managers, supervisors, therapists and family service coordinators, birth parents, resource parents and, when applicable, school personnel, state social workers and the child's attorney).<br>5. TST tools and assessments—KVC staff worked with TST developers to provide tools to help staff and foster parents apply their knowledge of TST into daily practice e.g., the Moment by Moment Assessments for Caregivers, the Emotional Regulation Guide, and the Priority Problem Worksheet. Also used a number of child assessment and wellbeing measures.<br>6. Community partners training—originally, TST training was provided for community partners on a child-specific basis to interested partners but over time this developed and in 2013, KVC provided its first formal training of community partners.<br>7. Birth parent training—Expanding TST knowledge and training to birth parents is the last step to full implementation. KVC has developed the birth parent curriculum with intended roll out in Autumn 2016.

Author/Year	Evaluation Design	Results and Limitations
Redd, Malm, Moore, Murphy and Beltz [27]	**Design:** process evaluation of program implementation (2012–2014) involving administrative data and qualitative research comprising 24 in-depth, semi-structured interviews with the KVC administrative leadership, directors, and managers; focus	**Findings:**<br>- In total, 384 KVC staff members were trained during the course of the first formal trainings; approximately 69% of KVC's 397 foster parents were trained over the course of the study period.

	groups with case managers (n = 30), therapists (n = 25), foster families (n = 40), family service coordinators (n = 20) and residential staff (n = 40).<br><br>**Measures:** researcher developed interview and focus group schedules; KVC and researcher developed TST fidelity measures used to assess staff fidelity to TST implementation on a quarterly basis.<br><br>.	- Across the interviews and focus groups that were conducted, KVC staff members reported that receiving multiple modes of training and repeated exposures to training was critical to successfully learning how to apply TST to their daily work.<br>- Staff valued the additional supports that were provided including professional role-specific workbooks, YouTube videos, email blasts to staff focused on specific TST topics, monthly staff and foster parent newsletters featuring articles on TST and "cheat sheets" (concise TST learning aids).<br>- Average TST "dosage" scores for each member of children's care-teams indicate that on average from 2012 to 2014 KVC staff implemented TST with increasing fidelity, with the average dosage score for children's care teams steadily increasing from 7.95 (SD = 2.25; out of 30) at the start of the roll-out (first quarter of 2012) to 20.77 (SD = 5.67) at the last quarter of 2014.<br>**Limitations:** Inability to randomly assign children to receive or not receive TST. The measure of TST dosage may not sensitively measure children's level of exposure to TST. Reliance on secondary data to measure all outcomes.
Murphy, Moore, Redd and Malm [23]	**Design:** Longitudinal quasi-experimental study using administrative data to evaluate impact of programme on children's well-being and placement stability. Follow up data collected through three years of TST (2011–2014).<br><br>**Measures:** Child functioning was assessed by children's caseworkers using the Child and Adolescent Functioning Assessment Scale (CAFAS; Hodges) on a quarterly basis (every 90 days); the Child Ecology Check-In (CECI) was used to assess children's emotional and behavioural regulation and was completed by children's caseworkers on a monthly basis; administrative placement history data were used to calculate children's placement stability; fidelity scores and TST training dates of children's care teams were used to calculate the level of TST or "dosage" that children received.	**Findings:**<br>- Increases in children's exposure to TST (overall dosage) were associated with significantly greater improvements in functioning and behavioural regulation.<br>- Increases in children's exposure to TST (overall dosage) were not associated with greater improvements in emotional regulation; however, higher levels of fidelity to TST in children's first quarter in KVC were associated with significantly greater improvements in emotional regulation.<br>- In addition, TST fidelity in children's first quarter in care, as well as increases in fidelity over time, were significantly associated with greater placement stability.<br>- Increases across quarters in inner circle dosage (those who worked most closely with the children) were associated with significant improvements in children's functioning and emotional regulation over time and increased placement stability.<br>- Outer circle members' implementation of TST in quarter one was significantly associated with improvements in functioning and placement stability.<br><br>**Limitations:** Inability to randomly assign children to receive or not receive TST. The measure of TST dosage may not sensitively measure children's level of exposure to TST. Reliance on secondary data to measure all outcomes.
**Country and Setting**	**TIC Approach and Components**	

Country—USA<br><br>Setting—Foster care<br><br>Developed by Anu Family Services, a treatment foster care agency that serves youth throughout Wisconsin and Minnesota	**Intensive Permanence Services (IPS)**<br><br>A trauma-informed model designed to help youth in out-of-home placement achieve permanency and strengthen their connections to supportive adults. IPS is delivered in four phases that take approximately 24 months, on average, to complete: Phase I—the Trusting Phase, Phase II—the Healing Phase, Phase III—the Connecting Phase and Phase IV—the Supporting Phase.<br><br>IPS involves:<br>• family search and engagement to identify and engage family members and other supportive adults who are important to the youth and may be willing to support the youth in their path to permanency;<br>• helping prepare youth for permanency by addressing trauma and psycho-education on grief and loss.<br><br>IPS focuses on the impact of trauma on the brain and the importance of supportive relationships, self-awareness, mindfulness and spiritual connections as core component of healing.	
**Author/Year**	**Evaluation Design**	**Results and Limitations**
Hall, Semanchin and Jones [24]	**Design:** Qualitative research to determine the key characteristics of IPS. How IPS workers experience implementing the model and barriers encountered.<br><br>**Measures:** staff survey consisting of open-response questions followed by semi-structured interview (n = 7).	**Findings:**<br>- Identified three overarching characteristics as key to the IPS model: (1) using a youth-driven approach; (2) having an organizational culture of well-being; and (3) promoting overall systems changes in work with children, youth, and families in child welfare.<br>- Identified barriers to implementing IPS: some foster parents, legal guardians, and potential connections were not prepared to deal with increases in the youth's pain-based behaviours during the healing process; carers' and referring workers' feelings of guilt, shame, fear and insecurity; youth who lacked the cognitive abilities or insight to address these past traumas and losses, or were otherwise unable to engage fully in the program.<br>- Noted that, of the young people who were involved in pilot project and completed at least 13 months, 80% (n = 20) achieved legal permanency while youth who were unable to complete IPS did not achieve legal permanency at this rate. Significant increase in scores on the Youth Connections Scale (YCS) from the time youth-initiated services to the time they were discharged.<br><br>**Limitations:** small sample, primarily self-reported
**Country and setting**	**TIC Approach and Components**	
Country—USA<br><br>Setting—Fostering/Adoption	ARC Model as part of wider **ADOPTS program**<br><br>• Implemented a 16-week structured application of the ARC treatment framework designed to be used as a brief outpatient intervention with adoptive children and their families which included:	

Services provided as part of the ADOPTS Program, a federally funded, multi-site treatment program established by Bethany Christian Services.	- 16 individual sessions and six group sessions for both children and caregivers.<br>- Weekly individual/family sessions which addressed clearly delineated treatment targets, with clinicians providing specific guidance as to session goals, psychoeducational content and intervention strategies.<br>• After receiving training in the model, clinicians received weekly supervision and monthly consultation, training and technical assistance from one of the treatment developers.<br>Clinicians filled out progress notes after each session, including ratings of how well they were able to implement the model's goals for that week, as well as the degree to which they needed to make modifications to the protocol.

Author/Year	Evaluation Design	Results and Limitations
Hodgdon, Blaustein, Kinniburgh, Peterson and Spinazzola [22]	**Design:** Pre/post-test evaluation of the application of the ARC model with pre-or post-adoptive children and carers who had two or more lifetime traumatic exposures, with current post traumatic stress disorder (PTSD) and functional impairment in two domains. Twelve month follow up.<br>**Measures:** Clinician Administered PTSD Scale (CAPS); Trauma Symptom Checklist for Children (TSC-C); Behavioral Assessment System for Children (BASC); Parenting Stress Index (PSI).	**Findings:**<br>- Significant lowering of Child Mental Health Symptoms with 76% of children assessed as having compared to 33.3% at follow-up.<br>- The effect size for the reduction in PTSD symptoms was large (Cohen's D = 1.88).<br>- Significant reductions were found for child anxiety, depression, posttraumatic stress, dissociationn and anger.<br>- Significant reduction in care-giver stress.<br>**Limitation:** lack of a control group, potential variability in treatment across clinicians, all evaluators were aware of treatment status of child
